# Molecular Insight into Ligand Binding and Transport by the Lentil Lipid Transfer Protein Lc-LTP2: The Role of Basic Amino Acid Residues at Opposite Entrances to the Hydrophobic Cavity

**DOI:** 10.3390/biom13121699

**Published:** 2023-11-24

**Authors:** Daria N. Melnikova, Ivan V. Bogdanov, Andrey E. Potapov, Anna S. Alekseeva, Ekaterina I. Finkina, Tatiana V. Ovchinnikova

**Affiliations:** 1 M.M. Shemyakin & Yu.A. Ovchinnikov Institute of Bioorganic Chemistry, the Russian Academy of Sciences, 117997 Moscow, Russia; contraton@mail.ru (I.V.B.); anna@lipids.ibch.ru (A.S.A.); finkina@mail.ru (E.I.F.); ovch@ibch.ru (T.V.O.); 2 Phystech School of Biological and Medical Physics, Moscow Institute of Physics and Technology, 141701 Dolgoprudny, Russia

**Keywords:** lipid transfer protein, Lc-LTP2, lentil, site-directed mutagenesis, lipid binding, lipid orientation, lipid transporting, lipid transfer mechanism

## Abstract

Lipid transfer proteins (LTPs) realize their functions in plants due to their ability to bind and transport various ligands. Structures of many LTPs have been studied; however, the mechanism of ligand binding and transport is still not fully understood. In this work, we studied the role of Lys61 and Lys81 located near the “top” and “bottom” entrances to the hydrophobic cavity of the lentil lipid transfer protein Lc-LTP2, respectively, in these processes. Using site-directed mutagenesis, we showed that both amino acid residues played a key role in lipid binding to the protein. In experiments with calcein-loaded liposomes, we demonstrated that both the above-mentioned lysine residues participated in the protein interaction with model membranes. According to data obtained from fluorescent spectroscopy and TNS probe displacement, both amino acid residues are necessary for the ability of the protein to transfer lipids between membranes. Thus, we hypothesized that basic amino acid residues located at opposite entrances to the hydrophobic cavity of the lentil Lc-LTP2 played an important role in initial protein–ligand interaction in solution as well as in protein–membrane docking.

## 1. Introduction

In plants, nonspecific lipid transfer proteins (LTPs) represent one of the most studied classes among other lipid-binding proteins. LTPs are small cationic proteins with a predominantly extracellular localization, which are able to bind and transport different ligands and thereby perform a variety of functions in plants. LTPs are essential for defense signaling [1], plant responses to biotic and abiotic stresses [2,3], seed development, germination, and lipid metabolism [4,5], as well as for deposition and function of wax- and lipid-based polymers (suberin and sporopollenin) [6]. They are also classified as allergens, and the formation of their complexes with ligands can affect the allergenic properties of LTPs. The study of molecular mechanisms of ligand binding and transport by LTPs is an important milestone, which can shed light on the biological functions of these proteins in plants and also gain a better understanding of sensitization and development of allergic reactions.

Most LTPs consist of a bundle of three or four α-helices linked by flexible loops and a stretch of C-terminal amino acid residues without any regular secondary structure. The LTP molecules contain a large central hydrophobic cavity where a variety of ligands can be accommodated [7]. The hydrophobic cavity has two entrances: the first one (termed as “top”) is narrower and located between helices H1 and H3 in the N-terminal part, and the second one (“bottom”) is restricted by the H2–H3 interhelical loop and the C-terminal tail. The cavity is considerably flexible, allowing these proteins to accommodate hydrophobic ligands of various shapes and sizes [8]. As shown earlier, both saturated and unsaturated fatty acids (FAs) as well as phospholipids can bind to plant LTPs and may be their natural ligands [7]. Inside the hydrophobic cavity, ligands can be accommodated in different orientations. It has been shown that FA may be located inside the cavity with its carboxyl group facing towards either the C-terminus or the N-terminus of the protein [9]. Moreover, two FA molecules may be positioned oppositely with carboxyl groups towards the C- and N-terminuses [10]. For lysophospholipids, a predominant location with the head phosphate group oriented towards the “top” entrance has been shown [11]. However, the opposite orientation of two LMPC molecules inside the hydrophobic cavity has also been observed in the case of wheat LTP1 [12]. These data indicate that FAs and other lipids can probably penetrate into the LTP molecules through both the “top” and “bottom” entrances of their hydrophobic cavities and be accommodated at different orientations there.

By now, little data are available regarding how plant LTPs bind and transport ligands, as well as amino acid residues playing key roles in these processes. It is worth noting that reported data have mainly been focused on the contribution of amino acid residues located near the “bottom” entrance of plant LTPs. Thus, it has been shown that Arg44 and Lys35 (numeration according to LTP1 from the Oryza sativa rice (PDB ID: 1RZL)) are involved in the interaction with lipids [13]. In some LTP1s, Tyr79 residue can form hydrogen bonds with the polar head group of lipid ligands [12,14]. In our previous work, we have shown that substitutions of Arg45 and Tyr80, located near the “bottom” entrance, affected the microarchitecture of the protein hydrophobic cavity, and reduced the effectiveness of FAs and lysolipid binding [15,16]. It has been assumed that the initial contacts of polar heads of FAs and lipids play an important role in the mechanism of ligand binding in solution. In NMR experiments, we have obtained data regarding the mechanism of lipid uptake by the lentil Lc-LTP2 from the surface of the LPPG micelle. We have assumed that the C-terminal end part of Lc-LTP2 acted as a membrane-binding domain and probably played an important role in the lipid uptake [17]. All data obtained did not provide a complete answer to the question: how do proteins of this class bind and transfer lipids?

Previously, we have shown that in the lentil Lc-LTP2 structure, not only does Agr45, in close proximity to the “bottom” entrance, have a positive charge, but also Lys81 and Lys92 [17]. Surprisingly, the “top” entrance of the hydrophobic cavity also included one basic residue, Lys61. Including that Lc-LTP2 most effectively binds negatively charged lysolipids and the described possibility of the lipid head orientation towards the “top” or “bottom” entrance of the LTP cavity, we hypothesized that positively charged amino acids located near both entrances to the hydrophobic cavity of the protein may participate in binding and transfer of ligands. In this study, we investigated a possible role of Lys61 and Lys81, located near the “top” and “bottom” entrances to the hydrophobic cavity of Lc-LTP2, respectively, in the binding of ligands in solution and in interaction with the membrane surface. For that purpose, using site-directed mutagenesis, we obtained mutants of Lc-LTP2—K61A, K81A, and K61A/K81A. By using fluorescence spectroscopy and molecular docking, we examined the ability of the protein-mutated variants to bind FAs and lysolipids. Using liposomes with calcein, as well as fluorescently labeled liposomes, we assessed the effect of the introduced substitutions on the ability of Lc-LTP2 to interact with model membranes and to transfer lipids, respectively.

## 2. Results

### 2.1. Molecular Docking

**Figure 1:**
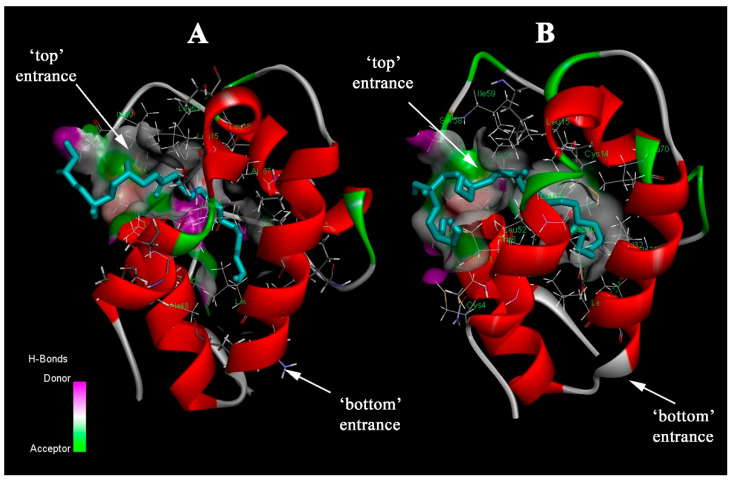
(**A**) Complex of wild-type Lc-LTP2 with LPPG solved by using NMR in solution (PDB: 2MAL); (**B**) complex of K61A mutated variant of Lc-LTP2 with LPPG (best conformation with a binding energy of −5.2 kcal mol^−1^), calculated by using computational molecular docking.

The orientation of the ligands (OLE, STE, LMPC, LMPG, LPPC, and LPPG) inside the protein hydrophobic cavity was investigated via computational molecular docking simulation. For each ligand and protein variant, 10 conformations of the ligand were calculated and analyzed. For all studied lysolipids (LMPC, LMPG, LPPC, and LPPG), orientation near the more hydrophobic “top” entrance of the tunnel and some part of the ligand exposed to the solvent was observed in all cases when the ligand was found in the hydrophobic cavity of the protein (Figure 1). The same orientation was observed in the NMR structure of the complex of wild-type Lc-LTP2 with LPPG in solution [17]. As for OLE and STE fatty acids, some conformations were found to be accommodated inside the hydrophobic cavity of wild-type Lc-LTP2 near the “top” entrance and some of them in the center of the tunnel, while in the case of mutated variants, they tend to be located only in a central position. The volume of the hydrophobic cavity did not undergo significant changes upon mutations: K61A did not lead to a change in the volume of the internal cavity volume, while K81A led to a decrease in the volume of <20 Å (with a 0.56 Å probe radius) from 832 Å in wild-type Lc-LTP2 to 813 Å in the case of K81A and K61A/K81A double-mutated variants. Neither K61A nor K81A were found to be participating in binding with FAs and lysolipids in molecular docking calculations. It is worth noting that molecular docking does not take into account initial contacts between a receptor (protein) and a target (ligand).

### 2.2. Competitive Binding Assay

**Figure 2:**
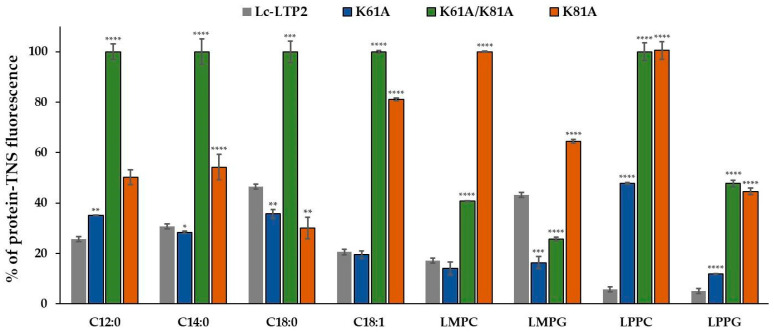
Effect of fatty acids and lysolipids on the fluorescence level of the protein-TNS complex. The results are expressed as the mean values (±SD) of the percentage of the fluorescence using the protein-TNS complex without ligand as a control. Data are representative of three experiments. Means with * (*p* < 0.05), ** (*p* < 0.01), *** (*p* < 0.005), and **** (*p* < 0.0001) are statistically significant compared with the fluorescence level of the Lc-LTP2-TNS complex.

To determine the effect of amino acid substitutions on Lc-LTP2 ligands binding in an aqueous solution, a TNS displacement assay was used (Figure 2). TNS is nonfluorescent in water, but fluoresces strongly in non-polar solvents or when bound to proteins. The binding of TNS to the LTP hydrophobic cavity resulted in an increase in fluorescence intensity, the value of which was taken as 100% for each protein. In this study, several groups of lipids were tested: (i) FAs (12–18 carbon atoms) having acyl chains of different lengths and degrees of saturation and (ii) lysolipids of different chain lengths (14–16 carbon atoms), namely lysophosphatidylcholine and lysophosphatidylglycerol. The choice of lipids was due to the fact that many of them are major in lentil seeds [18,19], and Lc-LTP2 has the highest binding ability with some of them [17]. No quenching between each of the ligands and TNS alone was detected, showing that these molecules did not interact directly.

The ability of the K61A mutant to bind lauric (C12:0, LAU), myristic (C14:0, MYR), stearic (C18:0, STE), and oleic (C18:1, OLE) acids was similar to that of Lc-LTP2 (65%, 45%, 64%, and 80% of the control fluorescence, respectively). K81A bound LAU, MYR, and OLE worse than Lc-LTP2 (50%, 46%, and 19% of the control fluorescence, respectively); however, binding capacities of STE were better than that of Lc-LTP2 (70% of the control fluorescence). More interesting results were obtained for K61A/K81A. Mutants did not bind with all tested FAs.

Slightly different results were observed in the case of lysolipids. For K61A, it was shown that the lysolipids reduced the fluorescence of the TNS-protein complex with different efficiency: 89% for LPPG, 53% for LPPC, 84% for LMPG, and 86% for LMPC. For K81A, TNS displacement efficiencies amounted to 36% for LMPG and 56% for LPPG. In the case of LMPC and LPPC, they did not influence the fluorescence of TNS at all. A more interesting result was obtained for K61A/K81A. This analog is associated with LMPG better than Lc-LTP2 (64% of the control fluorescence) and with LMPC and LPPG worse than Lc-LTP2 (60% and 53% of the control fluorescence). In the case of LPPC, the reduction in the K61A/K81A -TNS fluorescence was not observed, suggesting the absence of ligand binding with this mutant.

### 2.3. Liposome Leakage Assay

**Figure 3:**
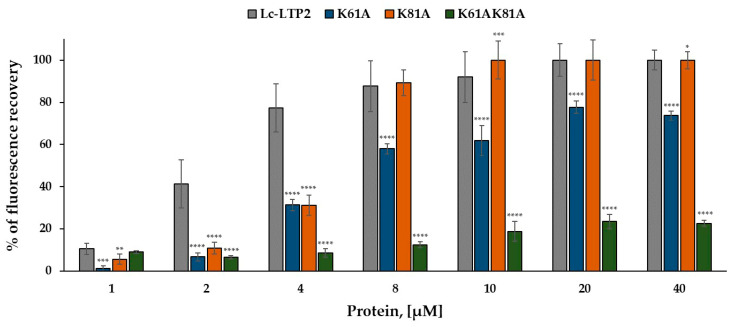
Percentage of calcein dye leakage from POPG SUVs upon addition of different concentrations of Lc-LTP2 and its mutants. Data are representative of three experiments, mean ± SD. Means with * (*p* < 0.05), ** (*p* < 0.01), *** (*p* < 0.005), and **** (*p* < 0.0001) are statistically significant compared with the data of Lc-LTP2.

This fluorescence leakage assay is a method for addressing membrane destabilization via biomolecules. Calcein-containing SUVs can be used for investigating different membrane-interacting proteins. Calcein leakage is a standard method for investigating membrane perturbations. Calcein is a water-soluble dye that is impermeable to lipid bilayers [20]. It self-quenches at concentrations of 60–100 μM and is pH sensitive [21]. For membrane permeabilization experiments, self-quenched calcein is encapsulated in lipid vesicles. If calcein leaks out, it is no longer quenched by other calcein molecules and its emission can be measured [21]. In this study, we used calcein-containing SUVs as a simple and intuitive method to investigate the ability of Lc-LTP2 mutants to interact with and degrade membranes in a protein-concentration-dependent manner. SUVs composed of zwitterionic (POPC) or anionic (POPG) phospholipids were tested. Effects of K61A, K61A/K81A, and K81A mutants on the permeability of the SUVs were investigated (Figure 3). All mutant proteins, like Lc-LTP2, did not release calcein from POPC vesicles. For POPG vesicles, it was shown that at small concentrations of 4 µM, all mutants were less effective than Lc-LTP2. At higher concentrations, 20 µM K81A, but not K61A, induced calcein leakage to the same extent as Lc-LTP2. K61A/K81A showed very weak vesicle-disrupting activity in all concentrations used.

### 2.4. Transfer of Lipids

Comparative studies of Lc-LTP2 and its mutants were investigated via fluorescence spectroscopy using labeled lipids. BODIPY fluorophores have been used to improve the performance of lipid transport assays because they have high stability in the environment, high photostability, low sensitivity to environmental polarity, and high emission intensity. Fluorescent lipid probe TMB-PC and its FRET quencher BCHB-PC were used for labeled POPC liposomes.

**Figure 4:**
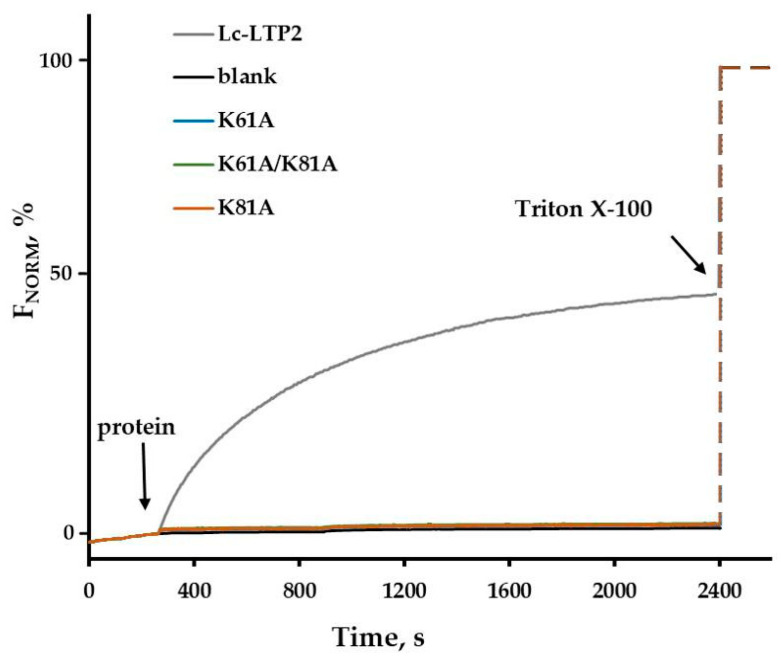
Assay of lipid transfer between the labeled (POPC) and unlabeled DMPG liposomes. The fluorescence intensity increased due to the transfer of lipid molecules between the liposomes upon the addition of the protein. The fluorescence intensity reaches a plateau when the fluorescent lipid molecules are equally distributed between the liposomes.

Two types of SUVs, the labeled POPC ones and the unlabeled DMPG liposomes, were mixed together. Before adding protein to the mixture, the fluorescence of TMB-PC was quenched. The addition of protein causes changes in TMB-PC fluorescence. For Lc-LTP2, we have previously shown that the fluorescence intensity increased steadily with time until a plateau was reached [17]. This illustrated the dilution of the labeled lipids in the liposomes due to the transfer activity of the protein. One possibility of the fluorescence increase is the transfer of TMB-PC or BCHB-PC to DMPG liposomes, which leads to labeled lipid dilution. In the case of K61A, K81A, and K61A/Y81A analogs, no change in TMB-PC fluorescence was observed, indicating that the proteins lack the ability to transport the tested lipids (Figure 4).

## 3. Discussion

A study of the structural features of LTPs, in particular, the mechanism of ligand binding to proteins, may provide some clues regarding the functional activities of these proteins. Despite the large number of studies that have been devoted to studying the interaction of LTP with lipids, this process is still obscure [7]. Often the term “nonspecific” is used in the names of LTPs, indicating that these proteins do not have one specific ligand and are capable of binding a wide range of lipids. However, different representatives of LTPs have a specificity in terms of preference in binding some ligands more effectively than others. For the tobacco LTP1 and the pea Ps-LTP1, lipid-binding specificity towards unsaturated FAs has been shown [22,23]. Recent studies on the *Juglans regia* walnut LTP1 have shown that this protein could bind exclusively to OLE [24].

Ligand binding in solution includes its uptake and retention inside the LTP hydrophobic cavity. In the case of the model or cell membranes, as assumed, protein-to-membrane docking, snatching of a lipid molecule, its penetration into the hydrophobic cavity, and correct orientation inside the protein take place. The size of the hydrophobic cavity and the amino acid residues lining its internal surface and located around its entrances play a key role in the initial protein–ligand interaction, ligand retention within the protein, and stabilization of the protein–ligand complex [7]. Alteration of the size and shape of the hydrophobic cavity, as well as replacement of key amino acid residues involved in various stages of the formation of the protein–ligand complex, may lead to changes in the specificity and efficiency of ligand binding and provide important information about the mechanisms of binding and transport of lipid ligands by plant LTPs. In the current study, we showed how the ability of lentil Lc-LTP2 to bind and transfer hydrophobic ligands changed when amino acid residues, presumably involved in the initiating step of ligand binding, were replaced. Basic amino acid residues—Lys61, located near the “top” entrance to the hydrophobic cavity, and Lys81, together with Arg45 and Lys92 located near the “bottom” entrance to the cavity—were chosen for these purposes. It is worth noting that the presence of charged amino acids near the entrances of the hydrophobic cavity is characteristic of the structures of many LTPs. The basic amino acid lysine at position 81, which is close to the “bottom” entrance, is quite typical for lipid transfer proteins. As for the “top” entrance to the hydrophobic cavity, many plant LTPs contain positively charged lysine residue and less frequently positively charged histidine and arginine residues at this protein region.

We obtained three mutant proteins of Lc-LTP2 (K61A, K81A, and K61A/Y81A) using site-directed mutagenesis. All the obtained mutants had a predominantly α-helical structure, similar to that of Lc-LTP2. Based on previous findings that the size and architecture of the hydrophobic cavity could influence the ability of a protein to bind lipids, we used computer simulations to investigate the size and volume of the hydrophobic cavity of the mutants of Lc-LTP2. We showed that the introduced substitutions almost did not affect the size and architecture of the hydrophobic cavity of all mutants.

In TNS displacement experiments, we investigated the ability of mutants to bind various FAs and lysolipids in solution. At the same time, computer simulations were used to evaluate the arrangement of ligands within the hydrophobic cavity and the role of various amino acid residues in stabilizing protein–ligand complexes. It was found that FAs, which are not those large molecules, could be accommodated in the hydrophobic cavity of the wild-type Lc-LTP2 in two different ways: some of them were found near the “top” entrance while the others were placed in the center of the tunnel. Interestingly, in the case of mono-mutated variants K61A and K81A, they were found only in the center of the hydrophobic cavity, but not near the “top” entrance. Neither K61A nor K81A mutants were found to be participating in binding with FAs in molecular docking calculations, which means that substituted amino acid residues, probably, do not participate in the stabilization of the complexes of Lc-LTP2 with FAs.

It was shown via fluorescence spectroscopy that single mutations of Lys61 or Lys81 did not greatly affect the efficiency of binding with all tested FAs; however, there was no binding with all tested FAs in the case of the double mutant K61A/K81A. This indicates that these two amino acid residues appear to be involved in binding with FAs. Taking into account all the data obtained, we assumed that FAs could enter from both entrances, and Lys61 and Lys81 initiated the formation of the complex with FAs, but the double substitution limited the access of FAs to both entrances to the hydrophobic cavity of the protein.

As in the case of our previously obtained NMR spectroscopy data for the Lc-LTP2-LPPG (complex (PDB ID: 2MAL), computational molecular docking showed that all the studied lysolipids inside the hydrophobic cavity of the protein and its mutants are located head up near the “top” entrance of the hydrophobic cavity. Analysis of amino acid residues involved in the binding of all studied lysolipids confirmed NMR data on the Lc-LTP2-LPPG complex and showed that Lys61 and Lys81 did not participate in the stabilization of protein–ligand complexes.

Using fluorescence spectroscopy, we found that the protein with the single Lys81 mutation was unable to bind uncharged lysolipids bearing the choline head group while it was still able to bind negatively charged lipids like LMPG and LPPG, but to a significantly lesser degree than the wild-type Lc-LTP2.

It can be assumed that disruption of the architecture of the “bottom” entrance in the K81A mutant significantly affected its binding with LMPC and LPPC, but the binding of negatively charged LMPG and LPPG was possible due to the presence of other positively charged amino acid residues there, namely Arg45 and Lys92. At the same time, Lys61 substitution led to a decrease in the ability to bind LPPC and LPPG, and to an increase in the efficiency of LMPC and LMPG binding, which have shorter acyl chains. Probably, in the latter case, the replacement of a positively charged amino acid residue with a hydrophobic residue can lead to a stronger retention of lysolipids with a short acyl chain in the protein hydrophobic cavity. The double-mutated protein K61A/K81A was able to bind LMPC, LMPG, and LPPG, but not LPPC. Based on the data obtained, we came to the conclusion that the binding of lysolipids in solution occurred through interaction with the “bottom” entrance and further penetration and location of the ligand inside the protein molecule with the polar head up.

To evaluate the effect of substitutions on the ability of the mutants to interact with model membranes, we used liposomes of different compositions filled with the fluorescent dye calcein. The mutants K61A and K81A at a concentration of 4 μM showed a similarly reduced ability to destabilize the membrane and caused 46% less calcein leakage compared with the wild-type protein. However, at a higher concentration of 20 μM, the difference is observed only in the case of the analog K61A, probably due to the presence of Arg45 and Lys92 near the “bottom” entrance to the cavity, compensating for the absence of Lys81. The ability of the double mutant to destabilize the membrane was extremely low at all concentrations used. We previously hypothesized that the surface accumulation of the lentil Lc-LTP2 destabilizes model membranes, ultimately causing transient membrane holes, rupture, and ultimately lysis. Based on the data obtained here, we assumed that the protein can have different orientations when interacting with the model membrane, contacting it through positively charged residues located at both the “top” and “bottom” entrances to the hydrophobic cavity. Therefore, both Lys61 and Lys81 substitutions make it difficult to retain the protein on the membrane, which causes only partial destruction of liposomes. Despite Lys61 and Lys81 taking part in the protein interaction with the liposome vesicles, they are probably not the most important residues in this process. We have previously shown that the substitution of Tyr80 resulted in the loss of the ability of Lc-LTP2 to destroy POPG liposomes. Probably, Tyr80 due to its aromatic ring is a key amino acid residue playing an essential role in Lc-LTP2-membrane docking [16].

We have previously shown that Lc-LTP2 could transfer lipids between POPC donor and DMPG acceptor liposomes [17]. In this study, we examined the role of amino acid residues Lys61 and Lys81 in the protein's ability to transfer lipids between membranes. For this purpose, we used the fluorescent lipid probe TMB-PC and its FRET quencher BCHB-PC. Surprisingly, all mutants studied in this work lost a lipid transfer ability. Previously, using NMR data on the LTP2-LPPG complex, we hypothesized that the protein interacted with the micelle via its “bottom” entrance and, after lipid snatching from the micelle, the penetration into the protein cavity took place. Then, the protein–ligand complex was formed, where the ligand head was located near the “top” entrance of the protein hydrophobic cavity. Taking into account that not only the K81A and K61A/K81A mutants but also the K61A mutant were not able to transfer lipids, we assumed that both entrances to the hydrophobic cavity of the proteins were involved in the lipid transfer process. Most likely, the amino acid residues Lys61 and Lys81, located near the opposite entrances to the hydrophobic cavity of the proteins, play an important role in the capture of lipids from the donor membrane and its expulsion from the protein cavity or its insertion into the acceptor membrane.

## 4. Materials and Methods

### 4.1. Materials

Synthetic phospholipids were purchased from Avanti Polar Lipids (Alabaster, AL, USA). FAs and 2-p-toluidinonaphthalene-6-sulphonate (TNS) were purchased from Sigma-Aldrich (St. Louis, MO, USA).

### 4.2. Cloning of Plasmids, Protein Expression, and Purification

Lc-LTP2 was produced as earlier described [25]. The K61A, K81A, and K61A/Y81A analogs were obtained via site-directed mutagenesis of the original plasmid pET-His8-TrxL-Lc-LTP2 using full-length inverse PCR amplification with mutagenizing primers (Supplementary Materials, Table S1). Plasmids containing DNA insert of alanine mutant proteins were transformed into E. coli BL21(DE3)-competent cells. Protein expression was induced using 0.1 mM of isopropyl β-d-thiogalactoside for 4 h at 37 °C. Purification of the recombinant proteins was carried out by using consecutive IMAC, dialysis, CNBr cleavage of the fusion protein, elimination of the carrier protein via subtractive IMAC, and final RP-HPLC.

### 4.3. Computational Molecular Docking

For computational calculations, spatial structure data from the NMR structure of lentil Lc-LTP2 in solution were used (PDB ID: 2MAL). Mutations K61A, K81A, and K61A/K81A were introduced into the structure of Lc-LTP2 using the mutagenesis wizard tool in PyMOL 1.8.2.0 software (Schrodinger, LLC, New York, NY, USA) [26]. The cavity volumes were calculated using the CASTp 3.0 online tool (http://sts.bioe.uic.edu/castp, accessed on 22 November 2023) with a 0.56 Å probe radius [27]. The 3D structure of oleic acid (PubChem ID: 445639) was taken from the PubChem database. Two-dimensional structures of stearic acid (PubChem ID: 5281), LPPC (PubChem ID: 460602), LPPG (PubChem ID: 86583416), LMPC (PubChem ID: 460604), and LMPG (PubChem ID: 118546616) were also taken from the PubChem database. Conversion of 2D structures into 3D space with the generation of optimal molecular geometry was carried out using the Open Babel v3.0.0 software [28]. Molecular docking of the target ligands with wild-type Lc-LTP2 and its mutated variants was performed with AutoDock Vina in the UCSF Chimera v.1.4 [29] using a Lamarckian genetic algorithm (LGA) for a blind ligand–receptor docking. Docking calculations were performed with default AutoDock Vina parameters in a user-defined grid box that covered all the volume of the protein tunnel. Docked structures were visualized with the ViewDock tool in the UCSF Chimera v.1.4 software. Analysis of amino acid residues participating in ligand binding was performed in Discovery Studio Visualizer v20.1.0.19295 [30].

### 4.4. Ligand Binding

A fluorescence-based ligand binding assay of Lc-LTP2 and its mutants (K61A, K81A, and K61A/Y81A) was carried out using TNS essentially as described previously [17]. Ligand binding analysis was performed at 25 °C. Fluorescence was measured at an excitation/emission wavelength of 320/437 nm with an F-2710 spectrofluorimeter (Hitachi High Technologies America Inc., Pleasanton, CA, USA). TNS (4 μM) with or without a ligand (4 μM) was incubated for 1 min in a stirred cuvette containing 2 mL of the 10 mM phosphate buffer (pH 7.4) with gentle mixing before the initial fluorescence (F0) was recorded. Then, proteins (4 μM) were added, and 2 min later the fluorescence was recorded at equilibrium (F). Experiments were performed in triplicate. The results were expressed as a percentage of the protein-TNS complex fluorescence calculated according to the formula [(F − F0)/FC] × 100%, where FC is the fluorescence of the protein-TNS complex in the absence of a lipid. Each experiment was performed in triplicate, independently. Data are expressed as means ± SD, which were calculated in all treatments using GraphPad Prism. Significant differences between means were analyzed via *t*-test.

### 4.5. Calcein Release Assay

For making the calcein-entrapped small unilamellar vesicles (SUVs) composed of POPC and POPG, a lipid film was rehydrated with PBS buffer containing 80 mM of calcein, pH 7.5. The liposome suspension was freeze-thawed for ten cycles and extruded ten times through a polycarbonate filter (100 nm pore size). Untrapped calcein was removed using gel filtration on a Sephadex G-50 column. The eluted calcein-entrapped vesicles were diluted to achieve the desired lipid concentration [31]. The emission difference between harvested and collapsed liposomes was evaluated before proceeding. Fluorescence measurements were performed at 20 °C on an F-2710 spectrofluorimeter (Hitachi High Technologies America Inc., Pleasanton, CA, USA). The wavelength emission and extinction were established at 535 and 485 nm, respectively. Liposomes which had at least 5-fold differences between assembled and destroyed liposomes were used in experiments. An assay on the ability of proteins to permeabilize the membrane was conducted in three replicates on a Plate Reader AF2200 (Eppendorf) at a wavelength of extinction and emission at 485 and 535, respectively. To compare the ability of proteins to degrade the membrane, 1% Triton solution was used as a positive control, and degradation was calculated using the formula: dye leakage (%) = (F − F0)/(Ft − F0) × 100%, where F0 and F are the fluorescence intensity before and after the protein addition, respectively. Each experiment was performed in triplicate, independently. Data are expressed as means ± SD, which were calculated in all treatments using GraphPad Prism. Significant differences between means were analyzed via *t*-test.

### 4.6. Lipid Transfer Assay

The fluorescence lipid transfer assay was performed using the fluorescent probe dilution method as previously described [17]. Briefly, two lipid probes, a fluorophore (1,3,5,7-tetramethyl BODIPY labeled phosphatidylcholine, TMB-PC) and its quencher (bis-cyclohexyl-BODIPY labeled phosphatidylcholine, BCHB-PC) were placed into SUVs [32]. The labeled vesicles (POPC, 30 μM) containing 0.8% TMB-PC and 1.6% BCHB-PC in 200 μL of the buffer containing 20 mM Tris, 1 mM EDTA, pH 7.4, were incubated with 6 μL of the unlabeled vesicle samples (1 mM DMPG in the same buffer). Then, 8 μL of the Lc-LTP2 (360 μM in the same buffer) was added to the mixture, and the variation in fluorescence intensity at 505 nm (excitation at 470 nm) was recorded as a function of time at 25 °C under constant stirring. Fluorescence was measured using an F-2710 spectrofluorimeter (Hitachi High Technologies America Inc., Pleasanton, CA, USA) with excitation and emission band passes of 2 nm and a stirred (∼100 rpm), temperature-controlled (25 °C), sample cuvette holder. Maximum fluorescence intensity (Ft) was determined by lysing vesicles in the presence of 1.5% Triton X-100. The obtained fluorescence intensity curves were normalized as follows:

Fnorm (%) = (F − F0)/(Ft − F0) × 100%, where F is the recorded fluorescence intensity and F0 is the fluorescence intensity measured immediately after the protein addition.

### 4.7. Dynamic Light Scattering

The mean hydrodynamic diameter of SUVs was measured via dynamic light scattering (DLS) using a particle size analyzer (Litesizer 500). All samples were diluted in PBS by a factor of 1:85. All determinations were made at 25 °C with a light incidence angle of 90°. The hydrodynamic diameter followed a Gaussian distribution and the polydispersity index was determined according to the width of particle size distribution. Major parameters included hydrodynamic diameter (<150 nm) and polydispersity index (<0.2).

## 5. Conclusions

The processes of ligand binding and transfer by LTPs include several stages. It is likely that different regions of plant LTP structures are responsible for the initial protein–ligand contact, ligand penetration into the protein cavity, its retention there, and subsequent release. In our study, we showed that Lys61 and Lys81, located on the surface of the lentil Lc-LTP2 near the “top” and “bottom” entrance of its hydrophobic cavity, respectively, are important for the binding of FAs and lipids in solution. Surprisingly, not only Lys81 but also Lys61 may take part in the protein interaction with model membranes according to the data obtained. Moreover, the presence of both Lys61 and Lys81 in the Lc-LTP2 structure is necessary for the transfer of lipids between membranes. Taking into account all the data obtained, we came to the conclusion that both amino acid residues participate in initial protein–ligand interactions in solution as well as in protein–membrane docking. Probably, electrostatic interactions of positively charged basic amino acids located at opposite entrances to the protein hydrophobic cavity with the negatively charged acidic groups of FAs and polar heads of other lipids play a key role in the formation of protein–ligand complexes and in the interaction of the protein with model membranes, which is important for the capture and release of the lipid molecules. The data obtained also demonstrates the possibility of modeling the specificity of plant LTPs, which opens up prospects for the application of these proteins in medicine, cosmetology, and agriculture since their ligands may be substances with different biological activities.

## Data Availability

No data are available in public data repositories. All data are available in this paper and supplementary materials.

## Supplementary Materials

The following supporting information can be downloaded at: https://www.mdpi.com/article/10.3390/biom13121699/s1, Table S1. List of mutagenizing primers.

## Abbreviations

LTP	Lipid transfer protein
LMPC	1-myristoyl-2-hydroxy-sn-glycero-3-phosphocholine
LMPG	1-myristoyl-2-hydroxy-sn-glycero-3-phospho-(1’-rac-glycerol)
LPPC	1-palmitoyl-2-hydroxy-sn-glycero-3-phosphocholine
LPPG	1-palmitoyl-2-hydroxy-sn-glycero-3-phospho-(1’-rac-glycerol)
POPC	1-palmitoyl-2-oleoyl-glycero-3-phosphocholine
POPG	1-palmitoyl-2-oleoyl-sn-glycero-3-phospho-(1’-rac-glycerol)
DMPG	1,2-Dimyristoyl-sn-glycero-3-phosphoglycerol
FA	Fatty acid
LAU	Lauric acid
MYR	Myristic acid
STE	Stearic acid
OLE	Oleic acid
TNS	2-p-toluidinonaphthalene-6-sulphonate
SUV	Small unilamellar vesicle
