# Supplementary material for: Molecular Insight into Ligand Binding and Transport by the Lentil Lipid Transfer Protein Lc-LTP2: The Role of Basic Amino Acid Residues at Opposite Entrances to the Hydrophobic Cavity
Source: Biomolecules. 2023 Nov 24;13(12):1699. doi: 10.3390/biom13121699 (PMC10741581; doi:10.3390/biom13121699)
Supplement: Supplementary file 1 [file biomolecules-13-01699-s001.zip › biomolecules-2726153-supplementary.pdf]

Supporting information

Molecular insight into ligand binding and transport by the lentil lipid transfer protein Lc-LTP2: the role of basic amino acid residues at opposite entrances to the hydrophobic cavity

Daria N. Melnikova [1,2,*], Ivan V. Bogdanov [1], Andrey E. Potapov [1,2], Anna S. Alekseeva [1], Ekaterina I. Finkina [1] and Tatiana V. Ovchinnikova [1,2]

1 M.M. Shemyakin & Yu.A. Ovchinnikov Institute of Bioorganic Chemistry, the Russian Academy of Sciences, 117997 Moscow, Russia; contraton@mail.ru (I.V.B.); anna@lipids.ibch.ru (A.S.A.); finkina@mail.ru (E.I.F.); ovch@ibch.ru (T.V.O.)

2 Phystech School of Biological and Medical Physics, Moscow Institute of Physics and Technology, 141701 Dolgoprudny, Russia

* Correspondence: d_n_m_@mail.ru.

Table S1. List of mutagenizing primers.

Mutation	Mutagenesis primer sequences (5′-3′)
sK61A	CAGCAGCCGGTTCTATTACTGTGTTGAATACTAACAACGCTGC
ansK61A	AGTAATAGAACCGGCTGCTGATT
sK81A	ATCAGTACCACCACCAACTGTAA
ansK81A	CAGTTGGTGGTGGTACTGATTGCGTAAGGAATGTTGACACCAC
